# Fears and pre-colonoscopy anxiety: a scoping review

**DOI:** 10.1590/0034-7167-2024-0618

**Published:** 2026-03-30

**Authors:** Hugo Castro, Nelson Martins, Patrícia Pontífice-Sousa

**Affiliations:** I Hospital da Luz Lisboa. Lisbon, Portugal; II Universidade Católica Portuguesa. Lisbon, Portugal; III Fundação Champalimaud. Lisbon, Portugal

**Keywords:** Fear, Emotion, Anxiety, Colonoscopy, Review., Miedo, Emoción, Ansiedad, Colonoscopia, Revisión.

## Abstract

**Objectives:**

to map the scientific evidence regarding the fears and emotions of patients undergoing colonoscopy, along with associated variables.

**Methods:**

a scoping review was conducted following the Joanna Briggs Institute Protocol, guided by the research question: “What are the fears and emotions associated with undergoing a colonoscopy, and associated variables?” A literature search was performed in the PubMed, CINAHL, Scopus, and RCAAP databases in June 2025, including articles in Portuguese, English, and Spanish.

**Results:**

a total of 512 references were retrieved. After applying the inclusion criteria, 24 articles published between 1990 and 2024 were analyzed. Reported fears and emotions included concerns about medical complications, diagnosis, and pain. Variables such as information about the procedure, previous experiences, and gender influenced anxiety levels.

**Conclusions:**

adopting a biopsychosocial approach reduces anxiety and improves both the quality and adherence to colonoscopy, guiding more effective and holistic care interventions.

## RESUMEN

**Objetivos:**

mapear la evidencia científica sobre los miedos y emociones de los pacientes sometidos a colonoscopia, así como las variables asociadas.

**Métodos:**

se realizó una revisión de alcance siguiendo el Protocolo del Instituto Joanna Briggs, con la pregunta de investigación: “¿Cuáles son los miedos y emociones asociados a la realización de una colonoscopia y variables asociadas?”. La búsqueda de artículos se llevó a cabo en las bases de datos PubMed, CINAHL, Scopus y RCAAP en junio de 2025, incluyendo publicaciones en portugués, inglés y español.

**Resultados:**

se obtuvieron 512 referencias. Tras aplicar los criterios de inclusión, se analizaron 24 artículos publicados entre 1990 y 2024. Los temores y emociones identificados incluyeron el miedo a complicaciones médicas, al diagnóstico y al dolor. Variables como la información sobre el procedimiento, experiencias previas y el sexo influyeron en los niveles de ansiedad.

**Conclusiones:**

la adopción de un enfoque biopsicosocial reduce la ansiedad y mejora tanto la calidad como la adherencia a la colonoscopia, orientando intervenciones de cuidado más eficaces e integrales

## INTRODUCTION

Life is characterized by experiences that generate a wide range of feelings and emotions, some associated with positive events, others with negative ones. Health-related events are typically linked to negative feelings and emotions that are inherent to the human condition. Endoscopic exams and procedures, particularly colonoscopy, are no exception. Colonoscopy is a common clinical procedure, widely recommended and used as part of colorectal cancer (CRC) screening strategies and for evaluating gastrointestinal symptoms.

Literature reviews support that CRC remains the second leading cause of cancer-related death in Portugal, the third in the United States, and the fourth in the United Kingdom. Regular screening exams are recommended for individuals at average risk between the ages of 50 and 75^(1)^. Among the recommended screening tests, colonoscopy is considered the gold standard^(2)^, as it enables the detection and removal of precancerous and cancerous lesions. Several studies and epidemiological research have shown associations between colonoscopy rates and CRC mortality, with an estimated prevention rate of up to 65%^(1)^. However, despite published recommendations, the number of screening colonoscopies remains below ideal levels^(1)^. The procedure is often described as an unpleasant short-term experience with long-term benefits that far outweigh the risks^(3)^. Given these findings, it is essential to understand why more people do not choose to undergo this important screening exam. This leads to the central question: “What are the fears, emotions, and anxiety associated with undergoing a colonoscopy, and what variables influence them?”a question that underpins and justifies such decisions.

The environment surrounding these procedures can be unfamiliar and distressing for both patients and their families. Existing studies on the fears and emotions experienced in this context do not offer a holistic view of the phenomenon, and no scientific mapping in this area has been identified. Therefore, it is crucial to map and systematize the evidence on the fears, emotions, and anxiety that patients experience, as this will help implement necessary interventions aimed at alleviating these feelings and improving adherence to screening colonoscopy^(1)^.

Fears related to the procedure^(1)^, the potential diagnosis, and even death when undergoing colonoscopy^(4)^, combined with patients’ preconceived notions based on their own knowledge or information obtained from family, friends, and the Internet^(5)^, can heighten anxiety and reduce the likelihood of accepting screening colonoscopy^(1)^, as well as negatively impact future experiences.

Cappell, a gastroenterologist at Royal Oak Hospital in Michigan, USA, notes that the personal experience and emotional, non-medical considerations of each member of the multidisciplinary team may play a role in understanding patients’ reluctance to undergo screening colonoscopy. In other words, if healthcare professionals reflect on their own experiences, feelings, fears, and anxieties regarding undergoing colonoscopy themselves, they may better understand how to address these emotional considerations with patients, therefore improving screening rates and delivering care that is more attuned to actual needs^(3)^.

The ideas described, along with the experience of caring for patients scheduled for colonoscopy, led us to question what is known about the fears and anxiety they experience, and the variables involved. Gaining a deep understanding of patients’ experiences can help stakeholders incorporate their perspectives into the development of resources and processes. This recognition is essential to improving the quality of colonoscopy and all related care. Based on this, conducting a literature review on this topic was deemed both useful and necessary, as although extensive research has confirmed that specific fears related to colonoscopy prevent patients from undergoing this screening exam, little is known about the nature of those fears^(1)^.

## OBJECTIVES

To map the scientific evidence on the fears and emotions experienced by patients undergoing colonoscopy, along with the associated variables.

## METHODS

The recommendations of the Joanna Briggs Institute (JBI) were followed, specifically using the PCC mnemonic: Population, Concept, and Context. Regarding the Population, it refers to patients undergoing colonoscopy. The Concept relates to fears, emotions, anxiety, and the variables that influence them. The Context concerns the pre-examination period of the colonoscopy.

The scoping review was conducted based on the methodology of the Joanna Briggs Institute (JBI)^(6)^ and in accordance with the PRISMA extension for scoping reviews (PRISMA-ScR)^(7)^. To streamline the research process, avoid duplication of reviews, enhance transparency, and reduce bias, a protocol was developed and duly published following JBI recommendations. The publication took place on the Open Science Framework (OSF) platform and was assigned the registration number doi.org/10.17605/OSF.IO/2ZDNT. This protocol also outlined the objectives of the review, the inclusion criteria, and the corresponding methods.

### Criteria for inclusion/exclusion

For the development of this scoping review, the recommendations of the Joanna Briggs Institute (JBI) were followed, specifically the PCC mnemonic: Population, Concept, and Context. Regarding the Population, the review included studies focusing on patients who were about to undergo colonoscopy, without applying any restrictions related to gender, ethnicity, or other personal characteristics. The only exclusion criterion applied at this stage was pediatric age. As for the Concept, the research focused on fears, emotions, anxiety, and the variables that influence them. In terms of Context, the review included studies that addressed only the pre-examination period of colonoscopy, excluding those in which the analysis of fears and concerns occurred during or after the procedure.

Quantitative, qualitative, and mixed-method studies, both primary and secondary, that addressed the research questions were considered. No restrictions were placed on the language of publication or the time frame. The only exclusion criterion was the inability to access the full text of the study.

### Research Strategy

An exploratory search was conducted in PubMed, JBI Evidence Synthesis, and the Open Science Framework to identify potential reviews on the same topic, and no similar studies were found. The search followed a three-phase structure. The first phase consisted of an initial search in the CINHAL Complete and PubMed databases to identify key studies and relevant search terms.

In the second phase, search terms were defined and a comprehensive search strategy was developed for the various databases and, when applicable, for grey literature. A complementary search was also performed during this phase to ensure the inclusion of studies that may not have been retrieved initially.

To locate studies for the review, searches were conducted in the following databases: MEDLINE via PubMed, CINAHL via the EBSCOhost platform, Scopus, Web of Science, and the Scientific Open Access Repository of Portugal (RCAAP). In each database, the initial Boolean phrase used was composed of free terms: “Fear* OR feeling* OR emotion* OR anxiety OR Anxious*” AND “colonoscopy”. Subsequently, based on these terms, an analysis of the respective indexed keywords was carried out and adapted to each database (Chart 1).

**Chart 1:** Research Strategy detailed by database

Databases	Searches	Expression	Results
CINAHL Complete (EBSCO Host)	S1	TI ( *Fear* ^*^ OR *feeling* ^*^ OR *emotion* ^*^ OR *anxiety* OR *Anxious* ^*^ ) OR MH ( *Fear* OR *Anxiety* )	141 271
	S2	TI *Colonoscopy* OR MH *Colonoscopy*	11 785
	S3 (S1 AND S2)	(TI (*Fear* ^*^ OR *feeling* ^*^ OR *emotion* ^*^ OR *anxiety* OR *Anxious* ^*^) OR MH (*Fear* OR *Anxiety*)) AND (TI *Colonoscopy* OR MH *Colonoscopy*)	140
PubMed	#1	((*Fear* ^*^[Title] OR *feeling* ^*^[Title] OR *emotion* ^*^[Title] OR *anxiety*[Title] OR *Anxious* ^*^[Title]) OR (Fear[MeSH Terms])) OR (*Anxiety*[MeSH Terms])	272 049
	#2	(*Colonoscopy*[Title]) OR (*Colonoscopy*[MeSH Terms])	39 717
	#3 (#1 AND #2)	(((*Fear* ^*^[Title] OR *feeling* ^*^[Title] OR *emotion* ^*^[Title] OR *anxiety*[Title] OR *Anxious* ^*^[Title]) OR (*Fear*[MeSH Terms])) OR (*Anxiety*[MeSH Terms])) AND ((*Colonoscopy*[Title]) OR (*Colonoscopy*[MeSH Terms]))	195
Scopus	1	( TITLE ( *fear* ^*^ OR *feeling* ^*^ OR *emotion* ^*^ OR *anxiety* OR *anxious* ^*^ ) AND TITLE ( *colonoscopy* ) )	79
Web of Science	1	TI=(*Fear* ^*^ OR *feeling* ^*^ OR *emotion* ^*^ OR *anxiety* OR *Anxious* ^*^) AND TI=(*Colonoscopy*)	97
RCAAP (B-ON)	S1	TI ( *Fear* ^*^ OR *feeling* ^*^ OR *emotion* ^*^ OR *anxiety* OR *Anxious* ^*^ OR ansiedade OR medo^*^ OR *sentiment* ^*^ OR emoção^*^ ) AND TI (*Colonoscopy* OR Colonoscopia)	717
	S2	*Restringir por: Fornecedor de Conteúdos:* RCAAP	1

### Studies’ selection

After completing the search, the studies retrieved were exported to the Rayyan platform, and duplicates were removed. Titles and abstracts of the identified studies were screened, excluding those that did not meet the inclusion criteria. Studies that fulfilled the selection criteria were retrieved in full and their complete texts were read to assess their relevance. It is important to note that the screening and analysis of the studies were conducted independently by the reviewers, and consensus was reached through discussion. Based on the nature of the scoping review and in accordance with JBI guidelines, no methodological quality assessment of the studies was performed.

### Data Extraction

To organize the data collected throughout the review process, as recommended for scoping reviews, the “Template for Study Details, Characteristics, and Results Extraction Instrument” provided by the Joanna Briggs Institute (JBI) was adapted for data extraction^(6)^. The data were described and recorded in a dedicated document created for this purpose using Microsoft Word, formatted as a table and adjusted to meet the reviewers’ needs, based on the contents of the aforementioned template.

As in the study selection phase, data from the articles to be included were extracted independently by both reviewers, and any disagreements were resolved through discussion. There was no need to contact the authors of the articles to request missing or additional data.

### Results’ Presentation

After extracting the data from the articles selected for the review, the results were presented in table format, highlighting the characteristics of the included studies. This was accompanied by a narrative summary that explores and explains how these studies are connected to the review’s objective and research question.

## RESULTS

### Included Studies

**Figure 1:**
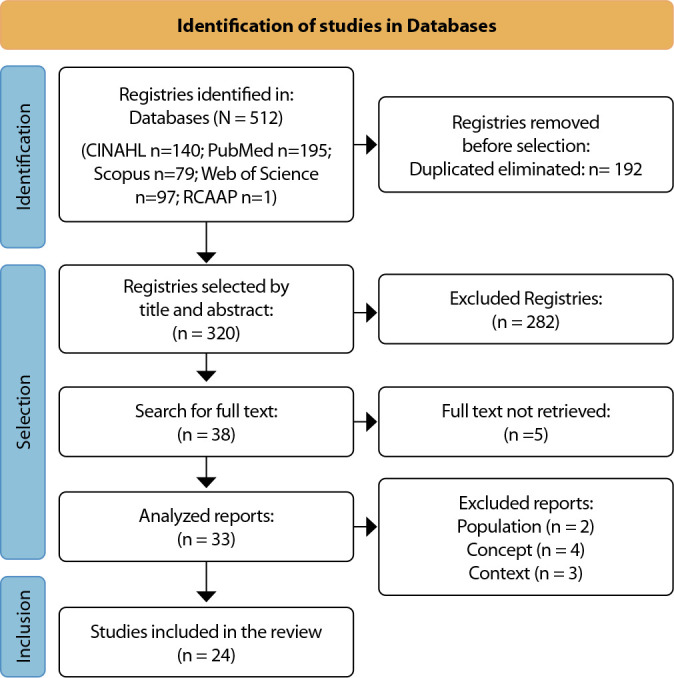
Studies identification and selected process *Preferred Reporting Items for Systematic Reviews and Meta-Analyses* (PRISMA) *diagram flow*

The objective of this scoping review was to map the scientific evidence on fears, emotions, and anxiety experienced by individuals undergoing colonoscopy, along with the variables that influence these experiences. To address this objective, the initial search yielded 512 articles. After removing duplicates, 320 remained. Following title and abstract screening, 282 articles were excluded in the first phase, leaving 38. Although full-text reading of all articles was intended, five could not be retrieved in full, resulting in 33 articles. In the second phase, after full-text review, nine were excluded for not meeting the inclusion criteria. Ultimately, 24 articles were included in the review, all of which addressed the research question. These studies were published between 1990 and 2024 and featured a variety of designs, including quantitative, qualitative, and mixed-method approaches, both primary and secondary, that responded to the research objectives (Figure 1).

**Figure 2:**
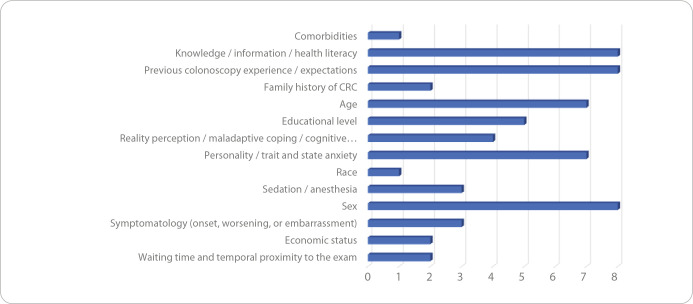
Fears, concerns, and emotions described in the analyzed articles, Lisbon, Portugal, 2025

**Figure 3:**
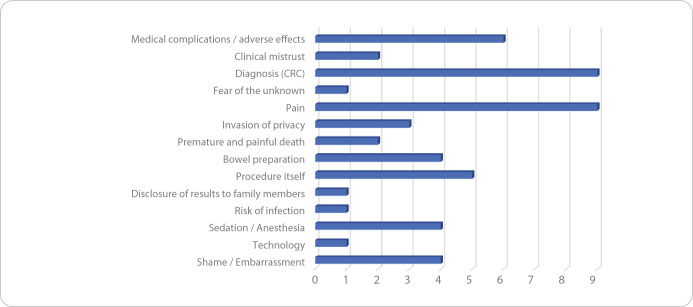
Variables influencing the fears, concerns, and emotions, Lisbon, Portugal, 2025

The characteristics and results of the included studies are presented in Chart 2. Charts 3 and 4 highlight the fears, emotions, and anxiety, along with the respective variables, as described in each of the 24 articles. Based on an analytical synthesis according to the frequency cited in the studies, fear of diagnosis appeared in 9 articles, fear of pain also in 9, and fear of medical complications/adverse effects in 6, making these the most frequently reported. Among the less frequently mentioned fears, emotions, and anxiety in the included literature were fear of premature and painful death (2 articles), fear of the unknown (1), disclosure of results to family members (1), and risk of infection (1) (Figure 2). A similar pattern was observed with the influencing variables. The most frequently cited were knowledge/information/health literacy (8 articles), previous colonoscopy experience/expectations (8), and gender (8). The least referenced variables were economic status (2 articles), family history of CRC (2), comorbidities (1), and race (1) (Figure 3)

**Chart 2:** Analysis of the selected articles, Lisbon, Portugal, 2025

	Title	Type and Study Design	Objective	Results
E1	Colonoscopy-specific fears in African Americans and Hispanics^(1)^	Questionnaire administered to 790 patients undergoing colonoscopy regarding demographic data, fears, and psychological variables.	Identify the presence of specific fears among African American and Hispanic individuals who underwent colonoscopy, and determine the factors associated with these fears.	Higher levels of fear: medical complications, pain, and CRC diagnosis Lower levels of fear: bowel preparation and sharing results with family members Female patients exhibited significantly higher levels of fear
E2	The Experience of Anxiety in Colonoscopy Outpatients^(2)^	Mixed, cross-sectional study. Questionnaire administered to patients awaiting colonoscopy or who recently underwent the procedure.	Determine the role of trait anxiety in patients’ situational anxiety to better understand their feelings toward colonoscopy.	Positive relationship between anxiety and maladaptive coping Previous experiences and unmet expectations increased anxiety levels
E3	It’s a Tube Up Your Bottom; It Makes People Nervous^(4)^	Prospective qualitative study. Exploratory interviews conducted with 13 patients who underwent an initial colonoscopy.	Prospectively consider the effect of colonoscopy on patients’ anxiety experiences at four time points related to an initial colonoscopy, using a qualitative approach.	Fears: embarrassment, diagnosis, and clinical mistrust
E4	Prospective study of anxiety in patients undergoing an outpatient colonoscopy^(8)^	Prospective study. Questionnaire administered to patients scheduled for colonoscopy to assess anxiety using a visual analog scale ranging from 0 to 100.	Determine the frequency and intensity of anxiety prior to a colonoscopy and the related factors.	Fears: diagnosis, complications, pain, and embarrassment Variables associated with increased anxiety: prior experience (first-time examination), gender (female), and age (younger patients)
E5	Is the choice of anesthesia during gastrointestinal endoscopic procedures a result of anxiety?^(9)^	Questionnaire administered to 723 patients undergoing elective endoscopy to collect sociodemographic data, medical history, and levels of state and trait anxiety.	Investigate the relationship between anesthesia preference during endoscopic procedures and anxiety levels.	Fears: pain, the procedure itself, and sedation Variables associated with higher anxiety: prior experience (first-time examination), absence of sedation/anesthesia, and temporal proximity to the procedure Fear of the procedure is one of the main reasons patients decline colorectal cancer (CRC) screening programs Patients who were sedated during a previous colonoscopy reported lower anxiety and greater acceptance when undergoing a new colonoscopy A significant increase in anxiety levels was observed prior to endoscopic procedures
E6	Fears of Having a Colonoscopy: Differences Between Veteran and Non-Veteran Patients^(10)^	Comparative study. Questionnaire administered to patients scheduled for colonoscopy between November 2014 and May 2016.	Identify the differences in perceived fears between veteran and non-veteran patients undergoing colonoscopy.	Fears: pain, complications, and bowel preparation Variable associated with increased anxiety: lack of knowledge
E7	*La importancia de informar ante la ansiedad y la resiliencia de pacientes que van a ser sometidos a una colonoscopia^(11)^*	Observational, descriptive, cross-sectional, and prospective study. Sample of 100 patients undergoing colonoscopy, with 50 receiving the procedure under conscious sedation and the other 50 under deep sedation.	Assess the level of anxiety and resilience in patients scheduled for colonoscopy and examine the influence of the knowledge and information they receive.	Fears: the unknown, pain, premature death, and diagnosis Implicated variables: knowledge, personality, and perception of reality Resilience also plays a role, defined as the ability to adapt to negative experiences, involving genetic, biological, psychological, and dynamic processes The more information patients receive, the lower their anxiety and the higher their resilience when facing a colonoscopy There is a negative correlation between resilience and anxiety
E8	Anxiety before gastrointestinal endoscopy-a significant problem?^(12)^	Questionnaire administered to 98 consecutive patients prior to undergoing upper and/or lower gastrointestinal endoscopy to collect data on anxiety, its causes, and possible interventions.	This study aimed to investigate the relationship between anesthesia preference during endoscopic procedures and anxiety levels.	Fears: the procedure itself, diagnosis, and anesthesia Variables associated with higher anxiety: limited information and lack of prior experience (first-time examination)
E9	Patient anxiety before invasive diagnostic examinations: coronarography, arteriography, and colonoscopy^(13)^	Observation of 93 patients to collect data related to anxiety using the State-Trait Anxiety Inventory and blood pressure assessment.	Assess the relationship between invasive diagnostic procedures and patients’ level of knowledge with their anxiety levels prior to undergoing such exams.	The authors conclude that the cognitive aspects of anxiety do not influence how patients experience physiological anxiety prior to invasive procedures
E10	*Qualidade dos cuidados em colonoscopia: ansiedade, dor, conforto e satisfação dos utentes^(14^* ^)^	Quantitative, descriptive, and correlational study. A questionnaire was administered to 60 patients scheduled for colonoscopy to collect data on anxiety levels, comfort, and satisfaction.	Determine the levels of anxiety, pain, comfort, and satisfaction in individuals undergoing colonoscopy.	Most influential fears on anxiety levels: sedation, test results, and potential complications Less influential fears on anxiety levels: embarrassment, technology, and invasion of privacy A moderate level of state anxiety is present before the procedure, which tends to decrease after its completion
E11	Who’s afraid of the big bad scope a study of fear^(15)^	Questionnaire administered to patients about the reasons behind their fear and anxiety prior to undergoing colonoscopy.	Assess the anxiety levels of patients scheduled to undergo colonoscopy.	High levels of fear and anxiety impact the patient’s experience and are associated with whether or not it is the patient’s first colonoscopy
E12	Anxiety Associated with Colonoscopy and Flexible Sigmoidoscopy: A Systematic Review^(16)^	Systematic review. A total of 58 studies were included, encompassing 24,490 patients between 2005 and 2017.	Assess the magnitude, types, and predictors of anxiety, as well as the corresponding interventions to reduce it, in patients undergoing colonoscopy or flexible sigmoidoscopy.	Fears: bowel preparation, the procedure itself, embarrassment, pain, complications, sedation, and diagnosis Variables associated with higher anxiety: female gender, elevated baseline anxiety, functional abdominal pain, low educational level, and lower socioeconomic status
E13	Unwillingness to participate in colorectal cancer^(17)^	Descriptive and correlational study. A cross-sectional, randomized telephone survey conducted with an ethnically diverse sample of adults aged 50 and older.	Identify the influence of medical mistrust, fears, attitudes, and sociodemographic characteristics on reluctance to participate in CRC screening.	Factors associated with reluctance to participate in CRC screening programs: embarrassment during the screening process, fear of contracting HIV, concern that the procedure may be painful, advanced age, fear of developing cancer, and medical mistrust
E14	Do difficulties in emotional processing predict procedure pain and shape the patient’s colonoscopy experience?^(18)^	Prospective, observational, and blinded study. Evaluation of 123 patients undergoing colonoscopy focused on specific emotional processing difficulties and variables related to anxiety.	Assess emotional processing difficulties and their related variables.	Emotional processing difficulties: unprocessed emotions, poor emotional regulation, avoidance of emotional triggers, and lack of emotional awareness Variables addressed: gender (higher anxiety levels in females), age (greater anxiety in younger individuals), prior colonoscopy experience (higher anxiety in first-time procedures), sedation (greater anxiety in non-sedated exams), anxiety, depression, and concern about the procedure Emotional processing difficulties assessed prior to endoscopy were positively correlated with behavioral manifestations of pain, self-reported pain, and pain catastrophizing An impoverished emotional experience reflects a deficit in emotional awareness, leading to increased pain sensitivity and severity through heightened anxiety and hypervigilance
E15	Psychological considerations in colonoscopy^(19)^	Scientific Article. Literature Review.	Analyze personality traits and other psychological influences that affect participation in screening examinations.	Fears: excessive fear of CRC, invasion of privacy, early and painful death Variables associated with higher screening adherence: female gender, Caucasian race, middle-class status, having first-degree relatives with CRC, onset, worsening, or embarrassment caused by symptoms, and knowledge about the screening procedure Positive correlation between personality traits and perceived vulnerability to developing cancer Belief in increased perceived vulnerability is correlated with age, and this heightened vulnerability contributes to greater participation in screening practices Higher screening adherence rates among women are linked to the belief that regular health maintenance reduces health problems
E16	The impact of a reminder preparatory instructions by phone on the anxiety state of patients undergoing elective outpatient endoscopy - a prospective randomized study^(20)^	Prospective randomized study. A random telephone survey was conducted with patients scheduled for colonoscopy to assess baseline anxiety levels.	Assess the impact of providing preparatory instructions for the procedure on anxiety levels.	Fears: pain, diagnosis, adverse effects Variables associated with higher anxiety: younger age, female gender, lack of prior experience (first-time procedure), and lower educational level
E17	Factors Associated with Anxiety About Colonoscopy: The Preparation, the Procedure, and the Anticipated Findings^(21)^	Survey administered to 1,316 patients scheduled for colonoscopy to collect data on patient characteristics and anxiety levels.	Assess the association between patient characteristics and anxiety related to bowel preparation, the procedure itself, and the expected outcomes of the examination.	Variables associated with increased anxiety: female gender, undergoing a first-time colonoscopy, bowel preparation, lack of information about the procedure, and presence of symptoms
E18	Common Anxieties of Patients Undergoing Oesophago-Gastro-Duodenoscopy, Colonoscopy and Endoscopic Retrograde Cholangio-Pancreatography^(22)^	Descriptive and correlational study. A questionnaire was administered to over 200 patients.	Investigate the relationship between anxiety levels in patients undergoing endoscopic examinations and their demographic characteristics, as well as their knowledge and understanding of the procedure.	Variables associated with higher anxiety: younger age and limited information about the examination
E19	Effects of patients’ anxiety, previous pain experience and non-drug interventions on the pain experience during colonoscopy^(23)^	Quantitative, cross-sectional, descriptive study. A questionnaire was administered to 130 patients undergoing colonoscopy to collect data on Trait-State Anxiety.	Evaluate patients’ anxiety levels prior to colonoscopy and identify correlations between anxiety levels and previous pain experiences.	Higher scores on the STAI scale were observed in individuals with high trait anxiety compared to those with state anxiety Female patients exhibited greater anxiety levels than male patients
E20	Anxiety Levels in Patients Undergoing Sedation for Elective Upper Gastrointestinal Endoscopy and Colonoscopy^(24)^	Survey of 500 patients undergoing upper gastrointestinal endoscopy and colonoscopy, focusing on demographic data and anxiety levels.	To evaluate the level of anxiety and its relationship with individual and social characteristics of patients undergoing upper gastrointestinal endoscopy and colonoscopy.	Fears: sedation According to the Beck Anxiety Inventory (BAI), higher anxiety levels were reported among the following variables: female gender, patients with comorbidities, and lower educational level
E21	A prospective randomized study to determine variables influencing anxiety level in patients undergoing colonoscopy^(25)^	Prospective, randomized study. A questionnaire was administered to 93 patients undergoing colonoscopy to assess both trait anxiety and state anxiety levels.	Determine the variables that influence anxiety levels in patients undergoing colonoscopy.	Variables associated with higher anxiety: lack of prior experience (first-time examination), limited information, lower educational level, and younger age Patients undergoing colonoscopy for the first time tend to exhibit higher anxiety levels, which generally decrease with repeated procedures Patients with higher education levels report lower anxiety
E22	Investigating knowledge gaps & level of anxiety of patient undergoing colonoscopy: a colonoscopy patient education questionnaire study^(26)^	Questionnaire administered to 42 patients regarding their education and knowledge about colonoscopy.	To evaluate the correlations between patients’ level of understanding and their degree of anxiety prior to undergoing colonoscopy.	Variables addressed: low levels of knowledge and educational needs 60% of patients presented with moderate to severe anxiety levels Half of the outpatients reported that they were well informed about the procedure
E23	The Effect of patient’s level of anxiety and knowledge on their experience of colonoscopy: a questionnaire survey^(27)^	Descriptive study. A questionnaire was administered over the course of one month to patients undergoing colonoscopy, aiming to collect data on demographic characteristics, clinical information, and anxiety levels.	To evaluate how patients’ pre-procedure anxiety and understanding of colonoscopy affect their post-procedure experience and satisfaction.	Variables associated with increased anxiety: Limited information about the procedure, leading to uncertainty and fear; Prolonged waiting time, which intensifies emotional distress and anticipation
E24	Anxiety and Health Literacy Levels of Patients Undergoing Colonoscopy^(28)^	Descriptive correlational study. A questionnaire was administered to 160 patients undergoing colonoscopy to collect: Identifying data, anxiety levels, and health literacy.	To determine anxiety levels and health literacy in patients scheduled for colonoscopy, as well as the variables that influence these outcomes.	Patients’ trait anxiety levels increase proportionally with their health literacy levels Anxiety drives health-seeking behavior and is associated with educational level and family history of cancer

**Chart 3:** Fears, concerns, and emotions described in the analyzed articles, Lisbon, Portugal, 2025

Study Fears, concerns, and emotions	1	2	3	4	5	6	7	8	9	10	11	12	13	14	15	16	17	18	19	20	21	22	23	24
Medical complications / adverse effects	X			X		X				X		X				X								
Clinical mistrust			X										X											
Diagnosis (CRC)	X		X	X			X			X		X	X		X	X								
Fear of the unknown							X																	
Pain	X			X	X	X	X					X	X	X		X								
Invasion of privacy										X			X		X									
Premature and painful death							X								X									
Bowel preparation	X					X						X					X							
Procedure itself				X	X				X			X		X										
Disclosure of results to family members	X																							
Risk of infection													X											
Sedation / Anesthesia					X					X		X								X				
Technology										X														
Shame / Embarrassment			X	X						X		X												

**Chart 4:** Variables influencing the fears, concerns, and emotions associated with colonoscopy, Lisbon, Portugal, 2025

Study Variables	1	2	3	4	5	6	7	8	9	10	11	12	13	14	15	16	17	18	19	20	21	22	23	24
Comorbidities																				X				
Knowledge / information / health literacy						X	X										X	X			X	X	X	X
Previous colonoscopy experience / expectations		X		X	X						X			X		X	X				X			
Family history of CRC															X									X
Age				X									X	X	X	X		X			X			
Educational level												X				X				X	X			X
Reality perception / maladaptive coping / cognitive aspects		X					X		X						X									
Personality / trait and state anxiety		X					X				X	X		X	X				X					
Race															X									
Sedation / anesthesia					X							X		X										
Sex	X			X								X		X		X	X		X	X				
Symptomatology (onset, worsening, or embarrassment)												X			X		X							
Economic status												X			X									
Waiting time and temporal proximity to the exam					X																		X	

## DISCUSSION

As previously mentioned, colonoscopy is considered the gold standard for the diagnosis and treatment of gastrointestinal diseases^(8,9)^. It is associated with a significant reduction in CRC mortality and is highly sensitive in detecting tumors, polyps, ulcers, active bleeding, and inflammatory disease^(10)^. However, achieving widespread adherence to colonoscopy remains a challenge^(10)^, as despite its clinical benefits, it is an uncomfortable procedure in which pain and fear of the unknown generate anxiety and stress in many patients^(9,11)^. Studies show that colonoscopy is often perceived negatively by society and commonly viewed as a source of anxiety and fear, which can prevent patients from undergoing the procedure and cause disruptions in the diagnostic and treatment process, contributing to the rejection of CRC screening programs^(9)^. This premise directly addresses the question: “Anxiety before gastrointestinal endoscopy - is it a significant problem?”^(12)^.

All the circumstances described above justify the need to analyze the frequency and intensity of anxiety caused by colonoscopy, as well as to identify the factors that contribute to this effect and their influence on procedural tolerance^(8)^. Although efforts have been made to improve tolerance through conscious or deep sedation, little attention has been paid to patients’ pre-procedural anxiety^(8)^. According to Cardenal, cited by Olmo-Conesa^(11)^, undergoing any diagnostic exam such as colonoscopy has emotional consequences for the patient, beginning at the time of scheduling^(13)^ and continuing through the procedure and the delivery of results. It is considered an acute situation that subsides once the triggering stimulus ends^(8)^. According to the study by Sequeira^(14)^, a moderate level of state anxiety was observed prior to the exam, which significantly decreased after the procedure, with statistical relevance.

Fear and anxiety, characterized by tension, nervousness, and worry^(8)^, are present in patients undergoing colonoscopy, and high levels of these emotions significantly affect the patient’s experience^(15)^. Several reasons are cited by patients for experiencing pre-endoscopic anxiety, including unpleasant experiences during previous endoscopies, alarm caused by personal research about the procedure, use of anesthesia^(12)^, bowel preparation^(16)^, potential diagnosis of a serious illness, complications during the procedure, fear of pain^(1,8)^, and feelings of embarrassment, shame, or invasion of privacy^(14)^. The factors most commonly associated with reluctance to participate in CRC screening programs include embarrassment, fear of contracting HIV, fear that the procedure may be painful, advanced age, fear of developing cancer, and medical mistrust^(17)^. According to the study by Miller et al.^(1)^, most of the participants reported specific fears related to colonoscopy which, although relatively low in intensity, were sufficient to prevent them from undergoing the screening exam.

Emotional processing difficulties assessed prior to colonoscopy were positively correlated with behavioral manifestations of pain, self-reported pain, pain catastrophizing, and the activation of emotional triggers^(18)^. An impoverished emotional experience reflects a deficit in emotional awareness, leading to increased sensitivity and severity of pain, proportional to heightened anxiety and hypervigilance^(18)^. A positive correlation was also observed between personality traits and vulnerability to cancer development^(19)^.

Several factors influence baseline anxiety levels, including age, sex, previous endoscopy experience, educational level, and concerns about adverse effects related to endoscopy, such as pain and worry about diagnosis^(20,21)^. Patients awaiting invasive procedures tend to exhibit higher rates of physiological anxiety, and the type of examination significantly affects their anxiety levels^(13)^. Higher state anxiety scores on the day of the endoscopy are significantly associated with lower educational levels, higher baseline anxiety, and lack of preparatory instructions^(20)^.

Studies indicate that younger patients^(22)^, females^(23)^, and those with comorbidities^(24)^ tend to present higher levels of anxiety, as do patients undergoing colonoscopy for the first time, although this tends to decrease with repeated procedures^(25)^. Other factors such as higher baseline anxiety, functional abdominal pain, lower educational attainment, and lower socioeconomic status have also been associated with increased anxiety prior to colonoscopy^(16)^. Research shows that the number of patients who experience anxiety before undergoing colonoscopy is alarming^(12,15)^ and highlights the need for renewed efforts and more assertive measures to help patients overcome their fear of the procedure and make this excellent diagnostic method more widely accepted, both by individuals and society at large^(12)^.

According to the study by Rollbusch^(2)^, there is a positive relationship between anxiety and maladaptive coping, as well as between previous experiences and unmet expectations. Meanwhile, the study by Wagner et al.^(19)^ indicates that adherence rates to screening exams vary according to several factors, including sex (higher participation among female patients due to the belief that maintaining health prevents future problems), race (greater participation among Caucasian individuals), economic status (higher screening rates among the middle class compared to higher or lower economic groups), having direct family members with CRC, and patients at high risk and/or with symptoms (such as sudden onset, increased severity, duration, or embarrassment). The belief in greater perceived vulnerability is correlated with age, and this increased vulnerability contributes to higher participation in screening practices^(19)^. On the other hand, excessive fear of CRC, the prospect of a premature and painful death, or the perception of an uncontrollable disease can trigger denial, in which the patient copes with fear by ignoring symptoms and resisting medical care, even when clearly necessary. These fears stem from cognitive, educational, and personality-related factors, as well as from the individual’s perception of reality and lack of accurate knowledge about CRC and its treatment. The combination of fear and lack of knowledge is a plausible explanation for the failure to participate in screening procedures, and it represents a challenge that must be addressed throughout life^(19)^.

Educational needs vary among patients, and more targeted and personalized information sessions are necessary^(26)^. The study by Hoang et al.^(27)^ demonstrates how patient anxiety and pre-procedural understanding/knowledge affect the post-colonoscopy experience and satisfaction. Patient anxiety levels are statistically related to the amount of information received, with the most resilient individuals being those who had or received more information about the procedure^(11)^. This is interpreted as anxiety experienced at a tolerable level that motivates individuals to seek health-related information^(28)^.

Meanwhile, the findings from Gebbensleben B et al.^(12)^ suggest that previous practices in managing fear before these procedures are highly questionable and indicate that new strategies are needed to effectively control fear and anxiety. It is therefore crucial for the healthcare team to be attentive to the mental and psychological state of the patients being examined^(15)^. Health initiatives should focus not only on increasing knowledge and addressing issues such as fear and mistrust, but also on normalizing CRC screening programs as a beneficial preventive practice^(17)^.

Patients should be supported in developing and using neutral language around colonoscopy, as this may help break the community taboo surrounding the procedure and intestinal health issues, potentially enabling better CRC prevention^(4)^. Given that colonoscopy is costly and invasive, and has the potential to cause harm, it is essential to clarify the importance of the nurse’s role in reducing patients’ anxiety during the experience^(4)^. According to Mateos, cited by Olmo-Conesa^(11)^, proper healthcare, specifically nursing care, goes beyond technical skills and is rooted in human care through a professional and comprehensive approach. It places the individual at the center of the entire care process, under a holistic view that encompasses all dimensions: biological, psychological, social, spiritual, and ecological.

A shift toward a biopsychosocial approach to healthcare and providing patients with a greater sense of control by involving them in decision-making, should be considered in gastroenterology centers, as it may have the potential to reduce anxiety^(4)^. According to studies, patients themselves suggest strategies to minimize and/or alleviate anxiety: the use of anesthesia, more detailed information about the procedures, a relaxed environment, the presence of a family member, and the option to watch the procedure on screen during non-sedated exams^(12)^. These findings should be taken into account when implementing measures to improve the quality and tolerance of colonoscopy^(8)^.

### Study limitations

The limitations of this study include potential language bias and the fact that only full-text articles were considered and included, which may have led to the exclusion of studies potentially relevant to the topic.

### Contributions to Nursing, Healthcare and Public Policy

Overall, these studies appear to align in recognizing the complexity and subjectivity of addressing such a broad topic, and they emphasize the need to expand research in this area with increasingly specific and targeted guidelines. Some studies suggest a correlation between higher educational levels, greater health literacy, and deeper knowledge with increased levels of fear and concern. Considering the rise of open access and the growing availability of information supported by artificial intelligence, it is projected that the patient of the future will be more educated and informed, posing new challenges for healthcare professionals within this evolving context. This new reality requires healthcare professionals to keep pace with these changes, seeking tools that provide the necessary skills to deliver optimal care to patients undergoing colonoscopy, and to learn from the outcomes.

It is important to clarify that the terms fear, concern, and anxiety are not limited to the perspective of diagnosis and/or cure but encompass multiple meanings and dimensions. Shifting this paradigm is essential to truly understand the other, and only through this lens can nursing care be delivered in its entirety. The contribution to the healthcare field lies in raising awareness of the need for professionals to reflect on their own experiences, emotions, fears, and anxiety regarding undergoing colonoscopy, in order to better understand patients and address their emotional concerns. The benefits are therefore widespread, for patients and families who receive care tailored to their real needs, and for all members of the multidisciplinary team, who not only benefit from new strategies aimed at improving screening rates but also experience personal fulfillment through the delivery of person-centered care.

## CONCLUSIONS

The literature increasingly supports that gastroenterology procedures are approaching the complexity of surgical interventions, despite their specific characteristics, where technical expertise is crucial and the focus lies in both theoretical and practical knowledge. However, it is equally essential not to overlook the importance of “knowing how to be”, understanding and empathizing with the person being cared for, recognizing what they are feeling, what they need, and how to enhance their comfort. This is especially relevant in the context of digestive endoscopy, where the patient’s time within the institution is limited and emotional levels are often heightened.

The analytical synthesis presented throughout this article allows the data obtained to be correlated with each professional’s personal clinical experience, linking the findings to insights gained over the course of their career. The perception of fears, particularly fear of diagnosis, pain, and medical complications/adverse effects, and the embedded variables such as knowledge/information/health literacy, previous colonoscopy experience/expectations, and gender, enable healthcare professionals to redirect care toward the actual needs of the population.

The dissemination of results from this scoping review aims to positively influence not only the nursing team but the entire multidisciplinary team involved in clinical endoscopic practice. These findings will foster greater awareness among all professionals of what patients experience throughout this process and offer practical applicability in care delivery. They also highlight the need and, above all, the relevance of intensifying and deepening research in such a subjective and vast area, supporting the conditions necessary to transform and improve the foundations and paradigms that underpin digestive endoscopy care. The concepts addressed are difficult to observe and verbalize, yet they represent a true challenge for healthcare professionals, serving as a catalyst for transcending the current situation and fostering personal growth. For some, fear can be a motivating force; for others, it may represent a significant barrier to screening.

Living in harmony with technological advancement requires a reinvention of thought, social interaction, empathy, and community, elements that distinguish humans from machines. It is crucial to reflect on empathy as a counterbalance to technology and its role in supporting and complementing the humanization of care. Healthcare professionals must have the humility to place themselves in the position of those being cared for, never relinquishing human creativity and perception, and must care with all three dimensions of knowledge - knowing, doing, and being - in their fullest expression. For all these reasons, in such a technical field, it is fundamental to understand what the other is feeling, aiming for greater “humanization of procedures”.

## Data Availability

The research data are available within the article.

## References

[1] Miller SJ, Iztkowitz SH, Redd WH, Thompson HS, Valdimarsdottir HB, Jandorf L. (2015). Colonoscopy-Specific Fears in African Americans and Hispanics. Behav Med.

[2] Rollbusch N, Mikocka-Walus AA, Andrews JM. (2014). The Experience of Anxiety in Colonoscopy Outpatients: A Mixed-Method Study. Gastroenterol Nurs.

[3] Cappell MS. (2011). Addressing unstated patient fears about colonoscopy to encourage reluctant patients to undergo screening colonoscopy. Gastrointest Endosc.

[4] Mikocka-Walus AA, Moulds LG, Rollbusch N, Andrews JM. (2012). “It’s a Tube Up Your Bottom; It Makes People Nervous”: the experience of anxiety in initial colonoscopy patients. Gastroenterol Nurs.

[5] Salmore RG, Nelson JP. (2000). The effect of preprocedure teaching, relaxation instruction, and music on anxiety as measured by blood pressures in an outpatient gastrointestinal endoscopy laboratory. Gastroenterol Nurs.

[6] Peters MD, Godfrey C, McInerney P, Munn Z, Tricco AC, Khalil H., Aromataris E, Lockwood C, Porritt K, Pilla B, Jordan Z (2024). JBI Manual for Evidence Synthesis.

[7] Tricco AC, Lillie E, Zarin W, O’Brien KK, Colquhoun H, Levac D, Moher D, Peters MDJ, Horsley T, Weeks L (2018). PRISMA Extension for Scoping Reviews (PRISMA-ScR): Checklist and Explanation. Ann Intern Med.

[8] Grilo Bensusan I, Herrera Martín P, Aguado Álvarez MV. (2016). Prospective study of anxiety in patients undergoing an outpatient colonoscopy. Rev Esp Enferm Dig.

[9] Erdal H, Gündoğmuş İ, Sinan Aydın M, Çelik B, Bolu A, Çelebi G, Serdar Sakin Y, Nuri Erçin C, Uygun A, Gülşen M. (2021). Is the choice of anesthesia during gastrointestinal endoscopic procedures a result of anxiety?. Arab J Gastroenterol.

[10] Paiji C, Kaye S, Yu AR, Reataza M, Jamal MM, Samarasena JB. (2016). Fears of Having a Colonoscopy: Differences Between Veteran and Non-Veteran Patients: 260. American Journal of Gastroenterology.

[11] Olmo-Conesa JM, Gómez-Díaz M. (2019). La importancia de informar ante la ansiedad y la resiliencia de pacientes que van a ser sometidos a una colonoscopia = The importance of reporting to the anxiety and the resilience of patients will undergo a colonoscopy. recs.

[12] Gebbensleben B, Rohde H. (2008). Anxiety before gastrointestinal endoscopy: is it a significant problem?. Dtsch med Wochenschr.

[13] Gryz J, Izdebski P. (2005). Patient anxiety before invasive diagnostic examinations : coronarography, arteriography, and colonoscopy. Pol J Radiol.

[14] Sequeira SR. (2016). Qualidade dos cuidados em colonoscopia: ansiedade, dor, conforto e satisfação dos utentes.

[15] Church J. (2017). Who’s afraid of the big bad scope? a study of fear and anxiety in patients awaiting colonoscopy. Dis Colon Rectum.

[16] Yang C, Sriranjan V, Abou-Setta AM, Poluha W, Walker JR, Singh H. (2018). Anxiety associated with colonoscopy and flexible sigmoidoscopy: a systematic review. Am J Gastroenterol.

[17] Bynum SA, Davis JL, Green BL, Katz RV. (2012). Unwillingness to participate in colorectal cancer screening: examining fears, attitudes, and medical mistrust in an ethnically diverse sample of adults 50 years and older. Am J Health Promot.

[18] Pontone S, Lauriola M, Palma R, Panetta C, Tomai M, Baker R. (2022). Do difficulties in emotional processing predict procedure pain and shape the patient’s colonoscopy experience?. BMJ Open.

[19] Wagner PJ, Kenrick JB, Rojas T, Woodward LD. (1995). Psychological considerations in colonoscopy. Prim Care.

[20] Hsu R, Kyo K, Maru S, Lee J, Leung J. (1999). The impact of a reminder preparatory instructions by phone on the anxiety state of patients undergoing elective outpatient endoscopy: a prospective randomized study. Gastrointest Endosc.

[21] Shafer LA, Walker JR, Waldman C, Yang C, Michaud V, Bernstein CN (2018). Factors associated with anxiety about colonoscopy: the preparation, the procedure, and the anticipated findings. Dig Dis Sci.

[22] Chuah SY, Goh KL, Wong NW. (1999). Common anxieties of patients undergoing oesophago-gastro-duodenoscopy, colonoscopy and endoscopic retrograde cholangio-pancreatography. Med J Malaysia.

[23] Ylinen E, Vehviläinen-Julkunen K, Pietilä A. (2009). Effects of patients’ anxiety, previous pain experience and non-drug interventions on the pain experience during colonoscopy. Journal of Clinical Nursing.

[24] Sargin M, Uluer M, Aydogan E, Hanedan B, Tepe M, Eryilmaz M (2016). Anxiety levels in patients undergoing sedation for elective upper gastrointestinal endoscopy and colonoscopy. Med Arh.

[25] Goenka P, Manalo G, Jobson B, Neumann J, Thomas E. (1999). A prospective randomized study to determine variables influencing anxiety level in patients undergoing colonoscopy. Gastrointest Endosc.

[26] Fan K, Siah K, Nianen M, Hasnah T, Chen Y, Anandraj S, Yan P. (2014). Investigating knowledge gaps & level of anxiety of patient undergoing colonoscopy: a colonoscopy patient education questionnaire study. J Gastroenterol Hepatol.

[27] Hoang N, Soubières A, Poullis A. (2016). The effect of patient’s level of anxiety and knowledge on their experience of colonoscopy: a questionnaire survey. Gut.

[28] Cevheroğlu S, Büyükyılmaz F. (2024). Anxiety and health literacy levels of patients undergoing colonoscopy. Gastroenterology Nursing.

