# Supplementary material for: Fears and pre-colonoscopy anxiety: a scoping review
Source: Rev Bras Enferm. 2026 Mar 30;79:e2020618. doi: 10.1590/0034-7167-2024-0618 (PMC13038224; doi:10.1590/0034-7167-2024-0618)
Supplement: Translation

# Translation: Medos e ansiedade pré-colonoscopia: uma revisão de escopo

**DOI:** 10.1590/0034-7167-2024-0618pt

**Authors:** Hugo Castro, Nelson Martins, Patrícia Pontífice-Sousa

**Affiliations:** I Hospital da Luz Lisboa. Lisboa, Portugal; II Universidade Católica Portuguesa. Lisboa, Portugal; III Fundação Champalimaud. Lisboa, Portugal

**Keywords:** Medo, Sentimento, Ansiedade, Colonoscopia, Revisão.

## Abstract

**Objetivos:**

mapear as evidências científicas sobre os medos e emoções dos pacientes submetidos à colonoscopia, e respectivas variáveis.

**Métodos:**

realizada uma *Scoping Review* segundo o Protocolo *Joanna Briggs Institute,* com a questão de pesquisa: “quais os medos e emoções associados à realização de uma colonoscopia e respectivas variáveis?”. Realizada pesquisa de artigos nas bases de dados PubMed, CINAHL, Scopus e RCAAP, em junho de 2025, com artigos em português, inglês e espanhol.

**Resultados:**

obtidas 512 referências. Após seleção de acordo com os critérios de inclusão, foram analisados 24 artigos, publicados entre 1990 e 2024. Nos receios e emoções incluem-se o medo de complicações médicas, diagnóstico e dor. Variáveis como informação do exame, experiências prévias e sexo influenciaram os níveis de ansiedade.

**Conclusões:**

a adoção de uma abordagem biopsicossocial reduz a ansiedade e melhora a qualidade e a adesão à colonoscopia, guiando uma intervenção de plenitude mais eficaz na prática dos cuidados.

## INTRODUÇÃO

A vida é caracterizada por experiências que geram múltiplos sentimentos e emoções, alguns associados a eventos positivos, outros a eventos negativos. Os eventos relacionados com a saúde estão normalmente associados a sentimentos e emoções negativas características do ser humano. Os exames e procedimentos de endoscopia, nomeadamente a colonoscopia, não são exceção. A colonoscopia é um procedimento clínico frequente, amplamente recomendada e utilizada como parte das abordagens de rastreio do cancro colorretal (CCR), e para avaliar sintomas gastrointestinais.

A pesquisa bibliográfica suporta que o CCR continua a ser a segunda principal causa de morte por cancro em Portugal, a terceira nos Estados Unidos e a quarta no Reino Unido, estando recomendado exames regulares de rastreio a pessoas de risco médio entre os 50 e 75 anos^(1)^. Dos exames de rastreio recomendados, a colonoscopia é considerada o padrão-ouro^(2)^ pois permite a deteção e a remoção de lesões pré-cancerígenas e cancerígenas, havendo já várias pesquisas e estudos epidemiológicos com associações entre as taxas de colonoscopia e a mortalidade por CCR, com uma estimativa de prevenção até 65%^(1)^. Mas, apesar das recomendações publicadas, o número de colonoscopias de rastreio permanece abaixo do ideal^(1)^, sendo referenciada como uma experiência desagradável de curto prazo com benefícios a longo prazo que excede em muito os riscos^(3)^. Mediante estes dados, é fundamental compreender o motivo pelo qual mais pessoas não optam por se submeterem a esse importante exame de rastreio e, desta forma, a pergunta que se impõe é “quais os medos/receios, emoções e ansiedade associados à realização de uma colonoscopia, e respectivas variáveis que interferem nos mesmos?”, que suportam e justificam essa decisão.

O ambiente que envolve estes procedimentos pode ser muito estranho, e uma experiência angustiante para o paciente e família. Os estudos sobre os medos e emoções vivenciados neste domínio não permitem uma visão holística do fenômeno, não tendo sido encontrado nenhum mapeamento científico nesta área. Desta forma, é crucial mapear e sistematizar a evidência sobre os medos/receios, emoções e ansiedade que os pacientes experienciam, pois tal contribuirá para implementar as intervenções necessárias, destinadas a amenizar os mesmos e melhorar a adesão à colonoscopia de rastreio^(1)^.

Os medos do procedimento^(1)^, do possível diagnóstico e de morte ao se submeterem ao procedimento^(4)^, aliados às ideias preconcebidas dos pacientes baseadas no seu próprio conhecimento, ou informações obtidas junto de familiares, amigos e Internet^(5)^, podem aumentar a ansiedade e diminuir a probabilidade de aceitarem ser submetidos à colonoscopia de rastreio^(1)^, além de prejudicar futuras experiências.

Cappell, Gastrenterologista do Hospital Royal Oak, Michigan, USA, refere que a experiência pessoal e as próprias considerações emocionais e não médicas de cada elemento da equipe multidisciplinar podem estar subjacentes para compreender a relutância do paciente em se submeter à colonoscopia de rastreio. Ou seja, se os próprios profissionais de saúde analisarem as suas experiências pessoais e os seus sentimentos, medos e ansiedade relativamente a serem submetidos à colonoscopia, vão entender melhor como abordar essas considerações emocionais junto dos pacientes para poderem assim melhorar as taxas de rastreio e prestar cuidados mais direcionados às reais necessidades^(3)^.

As premissas descritas e a experiência de cuidar de pacientes que vão ser submetidos à colonoscopia levaram-nos a questionar sobre o que se sabe dos medos e ansiedade que experienciam, e as variáveis implicadas. Obter uma compreensão profunda das experiências dos pacientes pode ajudar os intervenientes a incluírem a perspetiva dos mesmos no desenvolvimento de recursos e processos, sendo reconhecida a sua importância para melhorar a qualidade da colonoscopia e de todos os cuidados envolvidos. E foi nesta base que se considerou útil e fundamental a realização de uma revisão bibliográfica nesta temática, pois embora uma extensa pesquisa tenha confirmado que os medos específicos da colonoscopia impedem os pacientes de serem submetidos a este exame de rastreio, pouco se sabe sobre os mesmos^(1)^.

## OBJETIVO

Mapear as evidências científicas sobre os medos e emoções dos pacientes submetidos à colonoscopia, e respectivas variáveis.

## MÉTODOS

Foram utilizadas as recomendações do *Joanna Briggs Institute* (JBI), nomeadamente na mnemônica PCC: População, Conceito e Contexto. Relativamente à População, são os pacientes que são submetidos à colonoscopia. O Conceito é relativo aos medos/receios, emoções, ansiedade e respectivas variáveis que interferem nos mesmos. O Contexto diz respeito ao período pré-exame da colonoscopia.

A revisão *scoping* foi realizada com base na metodologia do JBI^(6)^ e de acordo com a extensão do PRISMA para revisões *scoping* (PRISMA-ScR)^(7)^. Para facilitar o processo de pesquisa, evitar a duplicação de revisões, aumentar a transparência e diminuir os vièses da investigação, foi elaborado um protocolo devidamente publicado, de acordo com as recomendações do JBI. A publicação decorreu na plataforma *Open Software Foundation* (*OSF*), tendo sido atribuído o número de registo doi.org/10.17605/OSF.IO/2ZDNT

Nesse protocolo foram igualmente explanados os objetivos da revisão, os critérios de inclusão e os respetivos métodos.

### Critérios de inclusão/exclusão

Para a realização desta revisão *scoping*, foram utilizadas as recomendações do JBI, nomeadamente a mnemônica População, Conceito e Contexto. Relativamente à População, a revisão incluiu estudos que incidiram sobre os pacientes que iriam ser submetidos à colonoscopia, não aplicando nenhuma restrição relativamente ao gênero, etnia ou outras características pessoais. Neste ponto aplicamos apenas o critério de exclusão de pacientes com idade pediátrica. Quanto ao Conceito, a pesquisa incidiu em medos/receios, emoções, ansiedade e respectivas variáveis que interferem nos mesmos. No Contexto, a pesquisa incluiu estudos que apenas abordassem o período pré-exame da colonoscopia, tendo como critério de exclusão o momento da análise dos medos e receios ser realizado durante ou após a realização da mesma.

Foram considerados os estudos do tipo quantitativo, qualitativo e misto, primários e secundários, que responderam às questões de investigação. Na pesquisa não foram definidas restrições quanto à língua em que o texto foi escrito nem limite temporal. Apenas foram restringidos estudos em que não foi possível obter o texto integral.

### Estratégia de pesquisa

Foi realizada uma pesquisa exploratória na *PubMed, JBI Evidence Synthesis* e *Open Science Framework* para identificar possíveis revisões com o mesmo tema, não tendo sido identificados trabalhos semelhantes. A pesquisa seguiu uma estrutura tripartida, sendo a primeira fase constituída por uma pesquisa inicial nas bases de dados *CINHAL Complete e PubMed*, de forma a identificar trabalhos-chave e os termos de pesquisa.

Na segunda fase, foram definidos os termos de pesquisa e construção da estratégia de pesquisa completa para as diferentes bases de dados e literatura cinzenta, quando aplicável. Realizada ainda uma pesquisa complementar à segunda fase para garantir a inclusão de estudos que possam não ter sido recuperados.

Para a localização dos estudos para a revisão, foi realizada pesquisa nas bases de dados *MEDLINE* via *PubMed* e *CINAHL* através da plataforma *EBSCOhost, Scopus, Web of Science* e Repositório Científico de Acesso Aberto de Portugal. Em cada base de dados foi primeiramente utilizada a frase booleana constituída pelos termos livres “Fear* OR feeling* OR emotion* OR *anxiety* OR Anxious*” AND “*colonoscopy*”, e de seguida, a partir desses termos, realizada análise das palavras-chave indexadas respectivas e adaptadas para cada base de dados (Quadro 1).

**Quadro 1:** Estratégia de pesquisa detalhada por base de dados

Base de Dados	Pesquisas	Expressão	Resultados
CINAHL Complete (EBSCO Host)	S1	TI ( *Fear* ^*^ OR *feeling* ^*^ OR *emotion* ^*^ OR *anxiety* OR *Anxious* ^*^ ) OR MH ( *Fear* OR *Anxiety* )	141 271
	S2	TI *Colonoscopy* OR MH *Colonoscopy*	11 785
	S3 (S1 AND S2)	(TI (*Fear* ^*^ OR *feeling* ^*^ OR *emotion* ^*^ OR *anxiety* OR *Anxious* ^*^) OR MH (*Fear* OR *Anxiety*)) AND (TI *Colonoscopy* OR MH *Colonoscopy*)	140
PubMed	#1	((*Fear* ^*^[Title] OR *feeling* ^*^[Title] OR *emotion* ^*^[Title] OR *anxiety*[Title] OR *Anxious* ^*^[Title]) OR (Fear[MeSH Terms])) OR (*Anxiety*[MeSH Terms])	272 049
	#2	(*Colonoscopy*[Title]) OR (*Colonoscopy*[MeSH Terms])	39 717
	#3 (#1 AND #2)	(((*Fear* ^*^[Title] OR *feeling* ^*^[Title] OR *emotion* ^*^[Title] OR *anxiety*[Title] OR *Anxious* ^*^[Title]) OR (*Fear*[MeSH Terms])) OR (*Anxiety*[MeSH Terms])) AND ((*Colonoscopy*[Title]) OR (*Colonoscopy*[MeSH Terms]))	195
Scopus	1	( TITLE ( *fear* ^*^ OR *feeling* ^*^ OR *emotion* ^*^ OR *anxiety* OR *anxious* ^*^ ) AND TITLE ( *colonoscopy* ) )	79
Web of Science	1	TI=(*Fear* ^*^ OR *feeling* ^*^ OR *emotion* ^*^ OR *anxiety* OR *Anxious* ^*^) AND TI=(*Colonoscopy*)	97
RCAAP (B-ON)	S1	TI ( *Fear* ^*^ OR *feeling* ^*^ OR *emotion* ^*^ OR *anxiety* OR *Anxious* ^*^ OR ansiedade OR medo^*^ OR *sentiment* ^*^ OR emoção^*^ ) AND TI (*Colonoscopy* OR Colonoscopia)	717
	S2	Restringir por: Fornecedor de Conteúdos: RCAAP	1

### Seleção dos estudos

Após a realização da pesquisa, os estudos encontrados foram exportados para o programa *Rayyan*, tendo sido eliminados os duplicados. Foi realizada a leitura dos títulos e resumos dos estudos encontrados na pesquisa, eliminando aqueles que não cumpriam os critérios de inclusão. Os estudos que cumpriam os critérios de seleção foram recuperados na íntegra e lidos os seus textos completos para verificação da sua adequação. De salientar que a leitura/análise dos estudos foi realizada de forma independente pelos revisores, e o consenso foi alcançado através da discussão. Com base na natureza da revisão *scoping*, e seguindo as indicações descritas pelo JBI, não foi realizada a avaliação da qualidade metodológica dos estudos.

### Extração dos dados

No sentido de organizar os dados colhidos ao longo do processo de revisão, tal como sugerido para as revisões *scoping*, foi adaptado o “*template study details, characteristics and results extraction instrument*”, disponibilizado pelo JBI para a extração de dados^(6)^. Os dados foram descritos e registados em documento apropriado e criado para o efeito, em Microsoft Word, em formato de tabela, ajustado às necessidades dos revisores, tendo por base os conteúdos do *template* referido.

Tal como na etapa da seleção dos estudos, os dados dos artigos a incluir foram extraídos de forma independente pelos dois revisores, tendo as discordâncias sido resolvidas pelo método de discussão. Não houve necessidade de contactar os autores dos artigos para solicitar dados ausentes ou outros.

### Apresentação dos resultados

Após a extração dos dados dos artigos selecionados para a revisão, os resultados foram apresentados sob a forma de quadro, revelando as características dos estudos incluídos, acompanhados por um resumo narrativo que explora e explicita a forma como os mesmos estão conectados com o objetivo e com a questão de pesquisa.

## RESULTADOS

### Estudos incluídos

O objetivo desta revisão *scoping* foi mapear a evidência científica sobre os medos/receios, emoções e ansiedade na pessoa submetida a colonoscopia, e respectivas variáveis que interferem nos mesmos. Para responder a este objetivo, na pesquisa inicial foram encontrados 512 artigos, que depois de removidos os duplicados, restaram 320. Após a leitura do título e resumo, em uma primeira fase foram excluídos 282, ficando 38. Sendo pretendido realizar a leitura integral dos artigos, não foi possível recuperar o texto integral em 5 artigos, restando assim 33. Depois, na segunda fase, após leitura integral destes, foram excluídos 9 por não cumprirem os critérios de inclusão. Por fim, foram incluídos 24 artigos para a revisão, que respondem à questão de partida. Estes foram publicados entre 1990 e 2024, com desenhos variados, nomeadamente estudos do tipo quantitativo, qualitativo e misto, primários e secundários, que responderam às questões de investigação (Figura 1).

**Figura 1:** Processo de identificação e seleção dos estudos *Preferred Reporting Items for Systematic Reviews and Meta-Analyses* **(PRISMA)** *diagram flow*

As características e resultados dos estudos incluídos são apresentadas no Quadro 2. Nos Quadros 3 e 4 são evidenciados os medos/receios, emoções e ansiedade, e respectivas variáveis como descrito em cada um dos 24 artigos. Com base em uma síntese analítica de acordo com a respetiva frequência citada nos artigos, o medo do diagnóstico consta em 9 artigos, medo da dor igualmente em 9, e medo de complicações médicas/efeitos adversos em 6, tendo sido desta forma os mais citados. Na literatura incluída no estudo, os medos/receios, emoções e ansiedade menos referenciados foram o medo de morte precoce e dolorosa em 2 artigos, do desconhecido em 1, revelação de resultados a familiares em 1 e risco de contágio igualmente em 1 (Figura 2). O mesmo se verifica com as variáveis implicadas, em que as mais citadas foram o conhecimento/informação/literacia em saúde em 8 artigos, experiência prévia de colonoscopia/expectativas em 8, e sexo igualmente em 8. As variáveis menos referenciadas foram status econômico em 2 artigos, história familiar de CCR em 2, comorbilidades em 1, e raça igualmente em 1 (Figura 3).

**Quadro 2:** Análise dos artigos selecionados, Lisboa, Portugal, 2025

	Título	Tipo e desenho de estudo	Objetivo	Resultados
E1	*Colonoscopy-specific fears in African Americans and Hispanics* ^(1)^	Questionário a 790 pacientes submetidos a colonoscopia sobre dados demográficos, medos e variáveis psicológicas.	Identificar a existência de medos específicos em afro-americanos e hispânicos que realizaram colonoscopia, e identificar fatores associados a esses medos.	Maior nível de medo: complicações médicas, dor e diagnóstico de CCR; Menor nível de medo: preparação intestinal e revelação de resultados a familiares; Doentes do sexo feminino apresentaram níveis de medos significativamente superiores.
E2	*The Experience of Anxiety in Colonoscopy Outpatients* ^(2)^	Estudo misto, transversal. Questionário entregue a doentes que aguardam colonoscopia ou submetidos à colonoscopia recente.	Determinar o papel da ansiedade-traço na ansiedade situacional dos doentes, para melhor entender os seus sentimentos em relação à colonoscopia.	Relação positiva entre ansiedade e enfrentamento desadaptativo; Experiências anteriores e expectativas não atendidas aumentaram níveis de ansiedade.
E3	*It’s a Tube Up Your Bottom; It Makes People Nervous* ^(4)^	Qualitativo prospectivo. Entrevistas exploratórias a 13 pacientes submetidos a uma colonoscopia inicial.	Considerar prospectivamente o efeito da colonoscopia nas experiências de ansiedade dos pacientes em 4 momentos relacionados a uma colonoscopia inicial, usando uma abordagem qualitativa.	Medos: constrangimento, diagnóstico, desconfiança clínica.
E4	*Prospective study of anxiety in patients undergoing an outpatient colonoscopy* ^(8)^	Prospectivo. Questionário entregue a pacientes que vão ser submetidos à colonoscopia, para avaliação de ansiedade através de escala visual analógica de 0 a 100.	Determinar a frequência e a intensidade da ansiedade antes de uma colonoscopia e fatores relacionados.	Medos: diagnóstico, complicações, dor e constrangimento; Variáveis abordadas para aumento de ansiedade: experiência prévia (exame inaugural), sexo (feminino) e idade (jovem).
E5	*Is the choice of anesthesia during gastrointestinal endoscopic procedures a result of anxiety?* ^(9)^	Questionário a 723 pacientes submetidos a endoscopia eletiva, para recolha de dados sociodemográficos, historial médico e níveis de ansiedade estado e traço.	Investigar a relação entre a preferência da anestesia durante procedimentos endoscópicos e os níveis de ansiedade.	Medos: dor, procedimento, sedação; Variáveis abordadas para maior ansiedade: experiência prévia (exame inaugural), sedação/ anestesia (sem), proximidade temporal do exame; O medo do procedimento é uma das razões mais importantes para os pacientes rejeitarem os programas de rastreio do CCR; Pacientes que foram sedados durante a colonoscopia anterior tiveram menor ansiedade e maior aceitação ao serem submetidos a nova colonoscopia; Verificado aumento significativo dos níveis de ansiedade antes dos procedimentos endoscópicos.
E6	*Fears of Having a Colonoscopy: Differences Between Veteran and Non-Veteran Patients* ^(10)^	Comparativo. Questionário realizado a pacientes agendados para colonoscopia (entre novembro de 2014 e maio de 2016).	Identificar as diferenças dos medos percebidos pelos pacientes veteranos e não veteranos submetidos à colonoscopia.	Medos: dor, complicações, preparação intestinal; Variável abordada para maior ansiedade: conhecimento (ausência).
E7	*La importancia de informar ante la ansiedad y la resiliencia de pacientes que van a ser sometidos a una colonoscopia* ^(11)^	Observacional, descritivo, transversal e prospectivo. Amostra de 100 pacientes submetidos à colonoscopia, sendo que 50 realizaram exame sob sedação consciente e os outros 50 sob sedação profunda.	Avaliar o nível de ansiedade e resiliência em pacientes que vão ser submetidos à colonoscopia e verificar a influência do conhecimento e informação recebidos.	Medos: do desconhecido, dor, morte precoce, diagnóstico; Variáveis implicadas: conhecimento, personalidade e percepção da realidade; A resiliência também desempenha um papel, definido como a capacidade de se adaptar a experiências negativas, que envolve processos genéticos, biológicos, psicológicos e dinâmicos; Quanto mais informação, menor a ansiedade e maior o nível de resiliência diante de uma colonoscopia; Existe uma correlação negativa entre resiliência e ansiedade.
E8	*Anxiety before gastrointestinal endoscopy-a significant problem?* ^(12)^	Questionário submetido a 98 pacientes consecutivos antes da realização de endoscopia digestiva alta e/ou baixa para recolha de dados sobre ansiedade, respectivas causas e intervenções.	Este estudo teve como objetivo investigar a relação entre a preferência da anestesia durante procedimentos endoscópicos e os níveis de ansiedade.	Medos: procedimento, diagnóstico, anestesia; Variáveis envolvidas para maior ansiedade: informação (limitada), experiência prévia (exame inaugural).
E9	*Patient anxiety before invasive diagnostic examinations: coronarography, arteriography, and colonoscopy* ^(13)^	Observação de 93 pacientes para coleta de dados relativos à ansiedade com a utilização da escala de Inventário de Ansiedade Estado e Traço e pela avaliação da pressão arterial.	Avaliar a relação do exame diagnóstico invasivo e o nível de conhecimento dos pacientes com os níveis de ansiedade antes da realização dos mesmos.	Os autores concluem que os aspetos cognitivos da ansiedade não influenciam a forma como os pacientes vivenciam a ansiedade fisiológica antes de exames invasivos.
E10	Qualidade dos cuidados em colonoscopia: ansiedade, dor, conforto e satisfação dos utentes^(14)^	Quantitativo, descritivo, correlacional. Questionário a 60 pacientes que vão ser submetidos à colonoscopia, para coleta de índices de ansiedade, conforto e satisfação.	Determinar os níveis de ansiedade, dor, conforto e satisfação das pessoas submetidas na colonoscopia.	Medos mais influenciadores nos níveis de ansiedade: sedação, resultado e complicações; Medos menos influenciadores nos níveis de ansiedade: vergonha, tecnologia e invasão da privacidade; Existência de um nível moderado de ansiedade-estado antes do exame, que diminui após a realização do mesmo.
E11	*Who’s afraid of the big bad scope a study of fear* ^(15)^	Questionário aplicado sobre o motivo do medo/ansiedade em pacientes prestes a realizar colonoscopia.	Avaliar os níveis de ansiedade dos pacientes que vão ser submetidos à colonoscopia.	Níveis elevados de medo e a ansiedade afetam a experiência do paciente e estão relacionados com o fato de ser ou não a primeira colonoscopia do paciente.
E12	*Anxiety Associated with Colonoscopy and Flexible Sigmoidoscopy: A Systematic Review* ^(16)^	Revisão sistemática. Incluídos 58 estudos, com um total de 24.490 pacientes, entre 2005 e 2017.	Avaliar a magnitude, tipos e preditores de ansiedade e respectivas intervenções de redução da mesma, em pacientes submetidos à colonoscopia ou sigmoidoscopia flexível.	Medos: preparação intestinal, procedimento, constrangimento, dor, complicações, sedação, diagnóstico; Variáveis implicadas para maior ansiedade: sexo (feminino), ansiedade basal aumentada, dor abdominal funcional, escolaridade (baixa) e estrato social (baixo).
E13	*Unwillingness to participate in colorectal cancer* ^(17)^	Descritivo, correlacional. Questionário telefônico transversal, e randomizado, realizado a uma amostra etnicamente diversa de adultos de 50 ou mais anos.	Identificar a influência da desconfiança médica, medos, atitudes e características sociodemográficas na relutância em participar no CCR.	Fatores associados à relutância em participar em programas de CCR: constrangimento durante o programa de rastreio, medo de contrair HIV, medo de que o exame possa ser doloroso, idade avançada, medo de desenvolver cancro e desconfiança médica.
E14	*Do difficulties in emotional processing predict procedure pain and shape the patient’s colonoscopy experience?* ^(18)^	Prospectivo, observacional, cego. Avaliação de 123 pacientes submetidos à colonoscopia sobre dificuldades específicas de processamento emocional e variáveis relacionadas à ansiedade.	Avaliar as dificuldades de processamento emocional e respetivas variáveis.	Dificuldades no processamento emocional: emoções não processadas, controle de emoções, evitar gatilhos emocionais, e falta de consciência emocional; Variáveis abordadas: sexo (maior grau de ansiedade no sexo feminino), idade (maior ansiedade nos mais jovens), experiência prévia de colonoscopia (maior ansiedade em colonoscopia inaugural), sedação (maior ansiedade em exames sem sedação), ansiedade, depressão e preocupação com o procedimento; As dificuldades de processamento emocional avaliadas antes da endoscopia foram positivamente correlacionadas com manifestações comportamentais de dor, dor autorrelatada e catastrofização da dor; Uma experiência emocional empobrecida corresponde a um déficit na consciência emocional, levando ao aumento da sensibilidade e gravidade da dor por meio do aumento da ansiedade e da hipervigilância.
E15	*Psychological considerations in colonoscopy* ^(19)^	Artigo científico. Revisão bibliográfica	Analisar variáveis de personalidade ou outras influências psicológicas que influenciam na participação de exames de rastreio.	Medos: “excessivo” de CCR, invasão de privacidade, morte precoce e dolorosa; Variáveis na maior adesão a exames de rastreio: sexo feminino, raça caucasiana, classe média, ter familiares diretos com CCR, aparecimento, agravamento ou constrangimento de sintomas, e conhecimentos sobre o exame; Correlação positiva entre a personalidade e a vulnerabilidade em relação ao desenvolvimento de cancro; A crença em maior vulnerabilidade percebida é correlacionada com a idade, e esse aumento na vulnerabilidade contribuiu para um aumento na participação nas práticas de rastreio; Taxa de adesão a exames de rastreio superiores no sexo feminino por convicção de que a manutenção diminui os problemas de saúde.
E16	*The impact of a reminder preparatory instructions by phone on the anxiety state of patients undergoing elective outpatient endoscopy - a prospective randomized study* ^(20)^	Prospectivo randomizado. Questionário telefônico aleatório para pacientes agendados para colonoscopia, para coleta dos níveis de ansiedade basal.	Avaliar o impacto de fornecimento de instruções preparatórias para o exame nos níveis de ansiedade.	Medos: dor, diagnóstico, efeitos adversos; Variáveis abordadas para maior ansiedade: idade (jovem), sexo (feminino), experiências anteriores (exame inaugural), nível de escolaridade (baixo).
E17	*Factors Associated with Anxiety About Colonoscopy: The Preparation, the Procedure, and the Anticipated Finding*s^(21)^	Questionário a 1316 pacientes que vão ser submetidos à colonoscopia para coleta de dados relativamente à características do paciente e ansiedade.	Avaliar a associação entre as características dos pacientes e a ansiedade relativa à preparação intestinal, ao procedimento e aos resultados esperados do procedimento.	Variáveis implicadas no aumento da ansiedade: sexo feminino, primeira colonoscopia, preparação intestinal, ausência de informação sobre o procedimento e sintomatologia.
E18	*Common Anxieties of Patients Undergoing Oesophago-Gastro-Duodenoscopy, Colonoscopy and Endoscopic Retrograde Cholangio-Pancreatography* ^(22)^	Descritivo, correlacional. Questionário entregue a mais de 200 pacientes.	Investigar a relação entre a ansiedade dos pacientes submetidos à exames endoscópicos, com as características demográficas e conhecimentos/ compreensão do procedimento.	Variáveis justificadas para maior ansiedade: Idade (jovem), informação sobre o exame (limitada).
E19	*Effects of patients’ anxiety, previous pain experience and non-drug interventions on the pain experience during colonoscopy* ^(23)^	Quantitativo, transversal, descritivo. Questionário a 130 pacientes submetidos a colonoscopia para coleta de dados de Ansiedade Traço-Estado	Avaliar a ansiedade dos doentes antes da colonoscopia e identificar correlações entre níveis de ansiedade e experiência prévia de dor.	Determinou-se maior pontuação na escala STAI em pessoas com alta ansiedade-traço do que em ansiedade-estado; Pacientes do sexo feminino são mais ansiosas do que do sexo masculino.
E20	*Anxiety Levels in Patients Undergoing Sedation for Elective Upper Gastrointestinal Endoscopy and Colonoscopy* ^(24)^	Questionário a 500 pacientes submetidos à endoscopia digestiva alta e colonoscopia, sobre dados demográficos e ansiedade.	Avaliar o nível de ansiedade, e a sua relação com características individuais e sociais dos pacientes que serão submetidos à endoscopia digestiva alta e colonoscopia.	Medos: sedação; De acordo com a escala Beck Anxiety Inventory (BAI), foram reportados níveis mais elevados de ansiedade em (variáveis): sexo feminino, pacientes com comorbilidades e nível educacional mais baixo.
E21	*A prospective randomized study to determine variables influencing anxiety level in patients undergoing colonoscopy* ^(25)^	Prospectivo, randomizado. Questionário entregue a 93 pacientes submetidos à colonoscopia. Para recolha do nível de ansiedade traço e estado.	Determinar as variáveis que influenciam o nível de ansiedade em pacientes submetidos à colonoscopia.	Variáveis abordadas para maior ansiedade: experiência prévia (exame inaugural), informação (escassa), escolaridade (baixa) e idade (jovem); Os pacientes submetidos à colonoscopia pela primeira vez apresentam níveis mais altos de ansiedade, mas isso tende a diminuir com a repetição dos procedimentos; Pacientes com ensino superior apresentam níveis mais baixos de ansiedade.
E22	*Investigating knowledge gaps & level of anxiety of patient undergoing colonoscopy: a colonoscopy patient education questionnaire study* ^(26)^	Questionário realizado a 42 pacientes sobre a sua educação/ conhecimentos em colonoscopia.	Avaliar as correlações entre o nível de compreensão e o grau de ansiedade antes da colonoscopia.	Variáveis abordadas: conhecimento/necessidades educacionais (baixos); 60% dos pacientes apresentavam níveis de ansiedade moderado/grave; Metade dos pacientes em ambulatório referem que estavam bem informados.
E23	*The Effect of patient’s level of anxiety and knowledge on their experience of colonoscopy: a questionnaire survey* ^(27)^	Descritivo. Questionário entregue a pacientes submetidos à colonoscopia (durante um mês) para coleta de dados demográficos, de informação clínica e de ansiedade.	Avaliar como a ansiedade e a compreensão pré-procedimento do paciente afetam a experiência e a satisfação pós colonoscopia.	Variáveis envolvidas para aumento de ansiedade: informação sobre o exame (limitada), tempo de espera (prolongado).
E24	*Anxiety and Health Literacy Levels of Patients Undergoing Colonoscopy* ^(28)^	Descritivo correlacional. Questionário a 160 pacientes submetidos à colonoscopia para coleta de dados identificativos, escala de ansiedade e escala de letramento em saúde.	Determinar a ansiedade e os níveis de letramento em saúde em doentes que vão realizar colonoscopia, e as variáveis que os afetam.	Os níveis de ansiedade-traço dos pacientes aumentam proporcionalmente com os níveis de letramento; A ansiedade motiva a pesquisa de saúde e está relacionada com nível educacional e histórico familiar de cancro.

**Quadro 3:** Medos/receios e emoções descritos nos artigos analisados, Lisboa, Portugal, 2025

Estudo Medos/receios e emoções	1	2	3	4	5	6	7	8	9	10	11	12	13	14	15	16	17	18	19	20	21	22	23	24
Complicações médicas/efeitos adversos	X			X		X				X		X				X								
Desconfiança clínica			X										X											
Diagnóstico (CCR)	X		X	X			X			X		X	X		X	X								
Do desconhecido							X																	
Dor	X			X	X	X	X					X	X	X		X								
Invasão de privacidade										X			X		X									
Morte precoce e dolorosa							X								X									
Preparação intestinal	X					X						X					X							
Procedimento				X	X				X			X		X										
Revelação de resultados a familiares	X																							
Risco de contágio													X											
Sedação/Anestesia					X					X		X								X				
Tecnologia										X														
Vergonha/constrangimentos			X	X						X		X												

**Quadro 4:** Variáveis que interferem nos medos/receios e emoções associados à colonoscopia, Lisboa, Portugal, 2025

Estudo Variáveis	1	2	3	4	5	6	7	8	9	10	11	12	13	14	15	16	17	18	19	20	21	22	23	24
Comorbilidades																				X				
Conhecimento/informação/letramento em saúde						X	X										X	X			X	X	X	X
Experiência prévia de colonoscopia/expectativas		X		X	X						X			X		X	X				X			
História familiar de CCR															X									X
Idade				X									X	X	X	X		X			X			
Nível de escolaridade												X				X				X	X			X
Percepção da realidade/enfrentamento desadaptativo/aspectos cognitivos		X					X		X						X									
Personalidade/ansiedade traço e estado		X					X				X	X		X	X				X					
Raça															X									
Sedação/anestesia					X							X		X										
Sexo	X			X								X		X		X	X		X	X				
Sintomatologia (aparecimento, agravamento ou constrangimento)												X			X		X							
Status econômico												X			X									
Tempo de espera e proximidade temporal para o exame					X																		X	

**Figura 2:** Medos/receios e emoções descritos nos artigos analisados, Lisboa, Portugal, 2025

**Figura 3:** Variáveis que interferem nos medos/receios e emoções, Lisboa, Portugal, 2025

## DISCUSSÃO

Como já referenciado, a colonoscopia é o padrão-ouro no diagnóstico e tratamento de doenças gastrointestinais^(8,9)^, associado a uma redução significativa na mortalidade por CCR e altamente sensível na identificação de tumores, pólipos, úlceras, hemorragia ativa e doença inflamatória^(10)^. No entanto, alcançar a adesão generalizada à colonoscopia tem sido um desafio^(10)^, pois mesmo sendo uma técnica benéfica, é um procedimento desconfortável, onde a dor e o medo do desconhecido geram ansiedade e estresse em muitos pacientes^(9,11)^. Estudos mostram que as colonoscopias em particular são percebidas de uma forma negativa pela sociedade e são comummente vistas como uma fonte de ansiedade e potenciadora de medo, que pode impedir o procedimento e causar interrupções no processo de diagnóstico e tratamento, contribuindo para a rejeição de programas de rastreio do CCR^(9)^. Esta premissa responde assim à questão se a “Ansiedade antes da endoscopia gastrointestinal - é um problema significativo?”^(12)^.

Todas as circunstâncias acima descritas justificam a necessidade de analisar a frequência e o grau de ansiedade que a colonoscopia provoca, bem como determinar os fatores que causam esse efeito e a sua influência na tolerância do procedimento^(8)^. Embora esforços tenham sido realizados para melhorar a tolerância usando sedação consciente ou profunda, pouca atenção tem sido dada à ansiedade pré-procedimento dos pacientes^(8)^. Segundo Cardenal, citado por Olmo-Conesa^(11)^, a realização de qualquer exame de diagnóstico como a colonoscopia tem efeitos prejudiciais ou negativos sobre o paciente a nível emocional, desde o momento do agendamento^(13)^ até ao resultado, incluindo durante o seu desenvolvimento, sendo uma situação aguda que desaparece quando o estímulo causador termina^(8)^. Segundo o estudo de Sequeira^(14)^ verificou-se a existência de um nível moderado de ansiedade-estado antes do exame, que diminui após a realização do mesmo, com relevância estatística.

O medo e a ansiedade caracterizados por tensão, nervosismo e preocupação^(8)^ existem em pacientes submetidos à colonoscopia, e altos níveis dessas emoções afetam a experiência do paciente^(15)^. São várias as razões que os pacientes referem para a presença de ansiedade pré-exame endoscópico, nomeadamente, experiências desagradáveis durante endoscopias anteriores, alarme com a pesquisa que realizaram sobre os exames, recurso à anestesia^(12)^, preparação intestinal^(16)^, diagnóstico potencial de uma doença grave, complicações durante o procedimento, medo da dor^(1,8)^ e constrangimento/vergonha/invasão de privacidade^(14)^. Os fatores mais associados à relutância em participar em programas de rastreio de CCR foram constrangimento, medo de contrair HIV, medo de que o exame possa ser doloroso, idade avançada, medo de desenvolver cancro, e desconfiança médica^(17)^. Segundo o estudo de Miller et al^(1)^, a grande maioria dos participantes referiram a presença de medos específicos na colonoscopia, que, apesar de serem em um nível relativamente baixo, eram o suficiente para impedir a realização do exame de rastreio.

As dificuldades de processamento emocional avaliadas antes da colonoscopia foram positivamente correlacionadas com manifestações comportamentais de dor, dor autorrelatada e catastrofização da dor, e com a ativação de gatilhos emocionais^(18)^. Uma experiência emocional empobrecida corresponde a um déficit na consciência emocional, levando ao aumento da sensibilidade e gravidade da dor, proporcional ao acréscimo da ansiedade e da hipervigilância^(18)^, verificando-se uma correlação positiva entre a personalidade e a vulnerabilidade em relação ao desenvolvimento de cancro^(19)^.

Vários fatores intervêm no estado de ansiedade basal, nomeadamente idade, sexo, experiência anterior de endoscopia, nível de escolaridade e preocupação com efeitos adversos relacionados à endoscopia como a dor e preocupação com o diagnóstico^(20,21)^, sendo que os pacientes que aguardam exames invasivos apresentam maiores taxas de ansiedade fisiológica, e que o tipo de exame influencia significativamente os seus níveis de ansiedade^(13)^. Scores mais altos de estado de ansiedade no dia da endoscopia estão significativamente associados a menor nível educacional, maior estado de ansiedade basal e ausência de instruções preparatórias^(20)^.

Estudos indicam que pacientes mais novos^(22)^, do sexo feminino^(23)^ e com comorbilidades^(24)^ apresentam níveis mais altos de ansiedade, bem como pacientes submetidos à colonoscopia pela primeira vez, mas que tende a diminuir com a repetição dos procedimentos^(25)^. Outros fatores como maior ansiedade basal, dor abdominal funcional, menor escolaridade e menor estrato social foram também associados à maior ansiedade antes da colonoscopia^(16)^. Os estudos mostram que o número de pacientes que sentem ansiedade antes de serem submetidos à colonoscopia é alarmante^(12,15)^, e permitem concluir que são necessários novos esforços e medidas mais energéticas para os pacientes superarem o medo do exame e tornar este excelente método de diagnóstico mais aceito pelos mesmos^(12)^ e pela sociedade em geral.

Segundo o estudo de Rollbusch^(2)^, existe uma relação positiva entre a ansiedade e o enfrentamento desadaptativo, e entre experiências anteriores e expectativas não atendidas. Por sua vez, o estudo de Wagner et al^(19)^ refere que a taxa de adesão a exames de rastreio varia de acordo com vários fatores, nomeadamente o sexo (pacientes do sexo feminino por convição de que a manutenção de saúde previne futuros problemas), raça (maior participação da raça caucasiana), status econômico (classe média tem maior percentagem de rastreio em comparação com o status econômico superior ou inferior), ter familiares diretos com CCR, e pacientes de alto risco e/ou com sintomatologia (início súbito dos sintomas ou aumento da sua gravidade, duração e constrangimento). A crença em maior vulnerabilidade percebida é correlacionada com a idade, e esse aumento na vulnerabilidade contribuiu para um aumento na participação nas práticas de rastreio^(19)^. Por sua vez, o medo excessivo de CCR, a perspectiva de uma morte precoce e dolorosa ou a visão de uma doença incontrolável pode desencadear negação, na qual o paciente lida com o medo, ignorando quaisquer sintomas que possa experimentar e resistindo aos cuidados médicos, mesmo que claramente necessário. Esses medos resultam de fatores cognitivos, educacionais, da personalidade, da própria percepção da realidade, e da falta de conhecimento correto sobre o CCR e o seu tratamento. A falta de conhecimento juntamente com o medo é uma explicação viável para a falha em participar em procedimentos de rastreio, sendo um problema que precisa ser abordado ao longo da vida^(19)^.

As necessidades educacionais diferem entre os pacientes e são necessárias sessões de esclarecimento mais direcionadas e personalizadas^(26)^. O estudo de Hoang et al^(27)^ demonstra como a ansiedade e a compreensão/conhecimento pré-procedimento do paciente afetam a experiência e a satisfação pós-colonoscopia. O nível de ansiedade dos pacientes relaciona-se estatísticamente com o nível da informação recebida, sendo os mais resilientes aqueles que apresentavam e/ou receberam mais informações sobre o exame^(11)^, e interpretado como a ansiedade experimentada em um nível tolerável que motiva e leva as pessoas a realizarem pesquisas de saúde^(28)^. Por sua vez, os resultados do estudo de Gebbensleben B et al^(12)^ fazem com que a nossa prática anterior de lidar com os medos antes destes exames pareça altamente questionável, e sugerem que o controle do medo e da ansiedade necessita de novas estratégias para ser alcançado. É assim crucial a equipe estar atenta ao estado mental e psicológico dos pacientes examinados^(15)^. Iniciativas de saúde devem concentrar-se não só no aumento do conhecimento e em abordar temas como medos e desconfiança, mas também normalizar os programas de rastreio do CCR como uma prática preventiva benéfica^(17)^.

Os pacientes devem ser apoiados no desenvolvimento e uso de uma linguagem neutra em torno da colonoscopia, pois pode conduzir à quebra do tabu da comunidade em torno do procedimento e dos problemas de saúde intestinal e, potencialmente, permitir uma melhor prevenção do CCR^(4)^. Como a colonoscopia é cara e invasiva, e tem o potencial de causar danos, é fundamental clarificar a importância do papel que o enfermeiro desempenha e deve desempenhar na redução da experiência de ansiedade dos pacientes^(4)^. Segundo Mateos, citado por Olmo-Conesa^(11)^, o cuidado adequado à saúde, especificamente o cuidado de enfermagem, não se esgota nas habilidades técnicas, mas baseia-se no cuidado humano a partir de uma abordagem profissional e de um ponto de vista abrangente, colocando a pessoa no centro de todo o processo de cuidado, sob uma visão holística que abrange todas as suas dimensões: biológica, psicológica, social, espiritual e ecológica.

Uma mudança em direção a uma abordagem biopsicossocial dos cuidados de saúde, e proporcionar aos pacientes um maior senso de controle ao participarem na tomada de decisões, deve ser avaliada nos centros de gastroenterologia, pois pode ter o potencial de reduzir a ansiedade^(4)^. Segundo estudos realizados, os próprios pacientes sugerem estratégias para minimizar e/ou dissipar a ansiedade: recurso à anestesia, mais informação detalhada sobre os procedimentos, um ambiente descontraído, presença de um familiar, assistir ao procedimento na tela em exames sem anestesia^(12)^, e estes achados devem ser levados em consideração na implementação de medidas para melhorar a qualidade e a tolerância da colonoscopia^(8)^.

### Limitações do estudo

Consideram-se limitações do estudo possíveis vièses de idioma e apenas terem sido considerados e incluídos artigos na integra, o que poderá ter conduzido à exclusão de algum potencialmente relevante para esta temática.

### Contribuições para a área da enfermagem, saúde ou política pública

Na sua generalidade, estes estudos parecem estar em consonância sobre a dificuldade de trabalhar um tema tão abrangente e subjetivo, e a necessidade de aumentar a investigação nesta área, com diretrizes cada vez mais específicas e direcionadas. Alguns estudos, por sua vez, defendem que existe uma correlação entre maior escolaridade, maior letramento em saúde e maior aprofundamento do conhecimento, com aumento dos níveis de medos e receios. Tendo em conta o acesso aberto e cada vez mais informação disponível com o apoio da inteligência artificial, projeta-se que o paciente do futuro seja uma pessoa mais formada e informada, e novos desafios para os profissionais de saúde com esta nova perspetiva. Esta nova realidade obriga que os profissionais de saúde consigam acompanhar esta tendência, que procurem ferramentas que forneçam as *skills* necessárias para proporcionar os melhores cuidados aos pacientes que irão ser submetidos a colonoscopia, e que aprendam com os respetivos *outcomes*.

Clarifica-se que os termos medo, receios e ansiedade não se limitam à perspetiva de diagnóstico e/ou cura, mas que apresentam múltiplos significados e dimensões. A mudança deste paradigma é fundamental para perceber o outro, e só desta forma é possível prestar cuidados de enfermagem na sua plenitude. A contribuição para a área da saúde reside na conscientização da necessidade dos profissionais de saúde analisarem as suas próprias experiências, sentimentos, medos e ansiedade relativamente a serem submetidos a colonoscopia para melhor entenderem o paciente e perceberem como abordar as suas considerações emocionais. Os benefícios são assim transversais a todos, para os pacientes e familiares que usufruem de cuidados mais direcionados às suas reais necessidades, e para todos os elementos da equipe multidisciplinar, que além de terem ganhos com as novas estratégias implementadas para a melhoraria das taxas de rastreio, sentem a realização pessoal de prestação de cuidados centrados na pessoa.

## CONCLUSÕES

A bibliografia suporta que cada vez mais, os procedimentos de Gastroenterologia estão muito próximos dos procedimentos cirúrgicos, apesar das especificidades que têm de ser levadas em consideração, onde a parte técnica é crucial e o foco reside no *saber-saber* e *saber-fazer*. Mas é também crucial não esquecer o *saber-ser*, o perceber e entender a pessoa que estamos a cuidar, o que está a sentir, o que precisa, como podemos aumentar o seu conforto, principalmente quando confrontados com a realidade da endoscopia digestiva em que o tempo que o paciente está na instituição é muito limitado, e as suas emoções estão normalmente em níveis mais elevados que o normal.

A síntese analítica apresentada ao longo do artigo permite relacionar os dados obtidos com a experiência pessoal de cada um na prática clínica, ou seja, correlacionar estes resultados com a experiência obtida ao longo da sua carreira. A percepção dos medos, nomeadamente do medo do diagnóstico, da dor e de complicações médicas/efeitos adversos, e das variáveis embutidas, como o conhecimento/informação/letramento em saúde, a experiência prévia de colonoscopia/expectativas e sexo, permitem ao profissional de saúde redirecionar os cuidados que presta às reais necessidades da população.

A divulgação dos resultados provenientes da revisão *Scoping* tem subjacente a intenção de influenciar positivamente não só a equipe de enfermagem, mas toda a equipe multidisciplinar envolvida na prática clínica dos procedimentos endoscópicos. Estes resultados permitirão a toda a equipe uma maior consciencialização do que o paciente sente ao longo deste processo, e uma aplicabilidade prática na prestação de cuidados. Evidencia igualmente a necessidade e principalmente a pertinência de intensificar e aprofundar a investigação em uma área tão subjetiva e vasta como esta, suportando condições para alterar e melhorar os alicerces e o próprio paradigma que suportam os cuidados específicos da área da endoscopia digestiva. Os conceitos abordados são de difícil observação e verbalização, mas constituem um verdadeiro desafio para o profissional de saúde, facilitador de transcendência da situação atual e de crescimento pessoal. Para alguns, o medo é um vértice motivador, mas para outros, pode representar uma barreira significativa para o rastreio.

Viver em equilíbrio com a evolução tecnológica exige uma reinvenção ao nível do pensamento, da socialização, da empatia e da própria comunidade, pois vai ser o que diferencia o ser humano das máquinas. É crucial refletir no foco da empatia como contraponto à tecnologia e na relevância de como pode apoiar e complementar a humanização de cuidados. O profissional de saúde tem assim de ter a humildade de se colocar no papel de quem está a ser cuidado, não prescindir da criatividade e percepção humana, e cuidar com os três saberes na sua verdadeira ascensão. Por tudo isto, em uma área tão técnica, é fundamental perceber o que o outro está a sentir, visando uma maior “humanização dos procedimentos”.

## Data Availability

Os dados de pesquisa estão disponíveis no corpo do artigo.
